# On the resolution of sexual conflict over shared traits

**DOI:** 10.1098/rspb.2024.0438

**Published:** 2024-07-31

**Authors:** Tanya M. Pennell, Judith E. Mank, Suzanne H. Alonzo, David J. Hosken

**Affiliations:** ^1^ Centre for Ecology & Conservation, Faculty of Environment, Science and Economy (ESE), University of Exeter, Cornwall Campus, Penryn TR10 9EZ, UK; ^2^ Department of Zoology and Biodiversity Research Centre, University of British Columbia, Vancouver, BC V6T 1Z4, Canada; ^3^ Department of Ecology and Evolutionary Biology, University of California, Santa Cruz, CA 95060, USA

**Keywords:** sexual conflict, intralocus conflict, resolution, dimorphism, sexual selection

## Abstract

Anisogamy, different-sized male and female gametes, sits at the heart of sexual selection and conflict between the sexes. Sperm producers (males) and egg producers (females) of the same species generally share most, if not all, of the same genome, but selection frequently favours different trait values in each sex for traits common to both. The extent to which this conflict might be resolved, and the potential mechanisms by which this can occur, have been widely debated. Here, we summarize recent findings and emphasize that once the sexes evolve, sexual selection is ongoing, and therefore new conflict is always possible. In addition, sexual conflict is largely a multivariate problem, involving trait combinations underpinned by networks of interconnected genes. Although these complexities can hinder conflict resolution, they also provide multiple possible routes to decouple male and female phenotypes and permit sex-specific evolution. Finally, we highlight difficulty in the study of sexual conflict over shared traits and promising directions for future research.

## 1. Introduction

Despite extensive work on the causes and fitness consequences of sexual conflict (differences in the evolutionary interests of the sexes [1–9]), many questions remain, particularly about the quantification and persistence of conflict. There are two general forms of sexual conflict. In one, shared traits that are genetically correlated between the sexes (where male and female relatives have correlated phenotypes because of shared genes) experience sexually antagonistic selection, with different trait values favoured in sperm and egg producers. In the second, fitness differences arise as a result of interactions between the sexes, with different interaction *outcomes* favoured in each. Here, we consider the current state of our understanding of the first type of conflict, with a focus on how it arises and what influences its resolution.

Terminology is key to conceptual frameworks, and the terms intralocus sexual conflict [3,5] and ontogenetic conflict [10] have both been used to describe conflict over shared phenotypes. The first term can be misleading, as it implies single locus effects on single traits, and perhaps biases our thinking towards simplistic resolutions. Many phenotypes are quantitative rather than Mendelian, and intralocus conflict does not fully capture this complexity. Ontogenetic conflict implicates whole developmental pathways and more complicated genetic architecture, which is more biologically realistic [11]. However, it also implies conflict during development, rather than between the sexes. In this review, we use the term ‘sexual conflict over shared traits’ to capture the conflict that arises due to sexually antagonistic selection –selection in opposing directions across the sexes –on traits with a shared genetic architecture. We note that this phrasing is not new (e.g. [12] and see [13]).

Sexual conflict arising from sexually antagonistic selection is as old as the evolution of the sexes. Isogamy without mating types is thought to be the ancestral gametic state [14], and subsequent disruptive selection on gamete size generated anisogamy and hence the sexes [15,16]. Anisogamy describes the universal difference between females (egg producers) and males (sperm producers) [15] and represents the initial form of sexual conflict, with disruptive selection for both small, motile gametes that ‘parasitise’ large, heavily provisioned gametes. The evolution of anisogamy itself therefore represents the resolution of an initial form of conflict over a shared trait, in this case gamete size, and opened the door to sexual conflict more broadly.

With gamete dimorphism, any gamete size/number trade-off [17] results in a greater abundance of sperm competing for limited eggs. This inevitably leads to sexual selection, and excess sperm generally results in sexual selection acting more strongly on males [4,18,19]. Sexual conflict follows, arising from sexually antagonistic (i.e. divergent sex-specific) selection on traits that have a shared genetic basis between the sexes (e.g. [2]). Thus, with the evolution of anisogamy, both sexual selection and conflict over shared traits are seemingly inevitable, except in idealized situations of strict genetic monogamy with no opportunity for extra-pair matings [20], or in those very rare cases where females and males do not share a genome (e.g. [21]). Sexually antagonistic natural selection also occurs, and although the relative contribution of natural and sexual selection to conflict over shared traits is unknown, sexual selection influences much sexual dimorphism and may play a proportionally larger role in generating conflict (but see [22–24].

Here, we explore how sexual conflict over shared traits may be weakened or resolved over evolutionary time. At one extreme, strong unresolved conflict occurs when males and females express the same trait values, despite strong sexually antagonistic selection. At the other extreme, complete resolution of conflict over shared traits would mean that both sexes express the phenotypes that maximize the fitness of each sex, and sexually antagonistic selection has ceased. It should also be noted that even when sexual conflict over the shared trait no longer exists, an inherent conflict between the sexes may be ongoing, e.g. females may still do better if males invested more in sperm size to permit the production of smaller more numerous eggs [25].

Although the full resolution of conflict over shared traits may be difficult because males and females in most species share nearly, or even, all of their genome (and sexual selection is ongoing), there are many phenotypic, genetic and genomic lines of evidence that suggest many individual cases of conflict over shared traits can be largely resolved. Most notably, i) phenotypic sexual dimorphism is indicative of at least partial resolution of conflict over shared traits, ii) artificial sex-specific selection can generate dimorphism [26], and iii) selection on traits in one sex need not always produce a correlated response in the other [27]. In addition, recent work has identified a myriad of routes to conflict resolution [11].

In this review, we draw upon the latest research and explore evidence for the resolution of conflict over shared traits and the extent to which genetic independence between the sexes can evolve. We comprehensively review the mechanisms through which resolution may occur, providing an update to earlier articles centred on this topic (e.g. [5,7]). We stress the multivariate nature of conflict, and finally, we suggest future directions of research.

## 2. Towards resolution

### (a) Phenotypic studies of conflict resolution

A trait may be implicated in conflict if there is sexually antagonistic selection acting on the trait, and the trait has an intersexual genetic correlation that deviates from 0 (on a scale of −1 to 1 [2]). Despite the (at times huge) differences between male and female phenotypes [28], the sharing of a genome means traits often have high intersexual genetic correlations, with some >0.8 [29], which may retard responses to sex-specific selection. However, sexually antagonistic selection is expected to break down genetic correlations to permit sex-specific evolution and therefore ameliorate conflict. Consistent with this, weakly negative associations between the degree of sexual dimorphism and the strength of male–female genetic correlations for individual traits have been detected [29–31]. In theory, weaker associations have either allowed more sexual divergence, or antagonistic selection has favoured weaker associations. Note that a negative intersexual genetic correlation for fitness can indicate strong conflict over shared traits, but there may be conflict over traits linked to fitness even when the intersexual genetic correlation for fitness itself is weak or positive [32]. Therefore, assessing selection on individual traits is crucial to our understanding of ongoing conflict.

**Figure 1.**
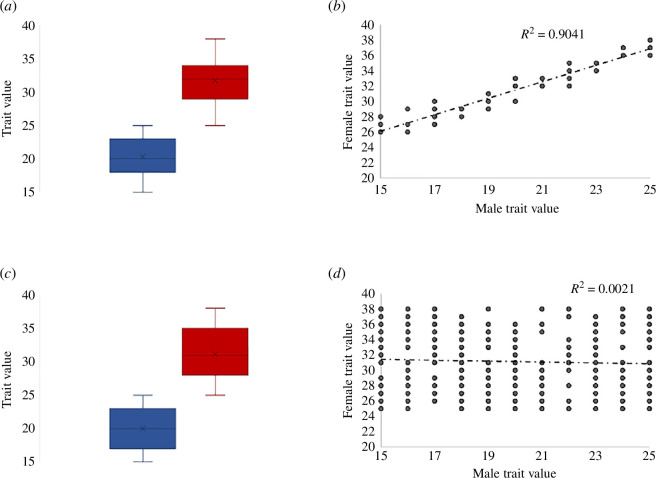
Sexual dimorphism can exist with high intersexual correlation. In this hypothetical example, mean shared trait values are largely non-overlapping between males (blue) and females (red), panels (*a*) and (*c*). However, the intersexual correlation (*r*_mf_) for a dimorphic trait, often measured by regressing average measurements of female versus male offspring within families, can vary. High *r*_mf_ in panel (*b*) is consistent with broad similarity in genetic architecture between males and females, with additional contribution from the sex determination pathway or sex hormones. Low *r*_mf_ in panel (*d*) is consistent with largely decoupled genetic architecture between the sexes.

The relationship between dimorphism and intersexual genetic correlation is complex, and therefore, dimorphism alone cannot predict how freely each sex may respond to selection or the extent to which conflict is resolved. For example, some studies have not recovered an association between intersexual genetic correlations for phenotypic traits and the degree of sexual dimorphism [33,34]. These studies suggest that for many traits (even those that are not obviously dimorphic), sex differences in genetic architecture, heritability, dominance or genetic variance exist [35]. These differences may provide routes to conflict reduction or resolution should these traits come under sexually antagonistic selection and may explain how genetic architecture can be rapidly decoupled under artificial selection. For example, intersexual genetic correlations of floral traits broke down after just five generations of artificial selection [36]. Furthermore, intersexual genetic correlations can be quite high for sexually dimorphic traits (see figure 1 for a hypothetical example). For example, human height, an obviously dimorphic trait, also exhibits a strong intersexual correlation and seems to be subject to sexually antagonistic selection [37]. In this example, trait genetic architecture permits at least partial sexual decoupling of height values, but each sex is not at its optima and conflict persists [37,38].

In addition, intersexual genetic correlations are often described by the term *r*_mf_, which considers genetic covariance between the sexes for a single trait. However, this may not be useful when predicting the evolution of sexual dimorphism and resolution of conflict when multiple traits covary [30,39], which we discuss in more detail below.

### (b) Conflict resolution for correlated phenotypes

Many phenotypes are characterized by genetically correlated suites of traits [40], and genetic covariances are as important as genetic variances in determining phenotypic evolution [41]. For example, measures of genetic dimensionality across a broad taxonomic range show that the amount of additive genetic variation underlying individual traits in univariate analyses is far less than what is available to selection when multiple traits covary [41]. This means that arguments describing individual character evolution in isolation are incomplete because when phenotypes are a web of correlated characters, selection on any trait can ripple through the phenotype to impact other traits in unpredictable ways (e.g. [42]). In addition, it means phenotypes are constrained to some degree by genetic covariances (as well as intersexual genetic covariances), as any individual trait will be linked to other traits. Thus, while considering intersexual genetic correlation for a single trait can reveal constraints, this will not capture the full inter-relatedness of male and female phenotypes [39].

The suite of genetic variances and covariances that underpin a phenotype is described by the genetic variance–covariance matrix, **G**. Furthermore, when considering the sexes and conflict over shared traits, estimates of the genetic variance–covariance matrix for each sex (**G**_F_ and **G**_M_: where the subscripts define female and male matrices), plus the multivariate intersexual covariances for all traits, **B**, are required. **B** is the multivariate analogue of *r*_mf_ and has been extensively discussed previously [2,30,39], so we only provide a short summary of how this can affect sexual conflict over shared traits.

Modelling by Lande [2] indicated how intersexual genetic correlations can cause selection on one sex to cause maladaptive changes in the other. However, although the maladaptive phase may only be transitory and not present at equilibrium, evolution towards equilibrium may require many generations, especially for traits with high intersexual correlation [2,29]. Lande’s model assumed no evolution of the genetic parameters and both a monomorphic **G** and symmetrical **B**. However, if **G** and **B** are not sexually symmetrical (not the same in both sexes or when transposed), the evolution of sex-specific phenotypes may be easier [39]. For example, if **G** is larger in one sex, then this sex will respond more to the same selection strength, generating sexual dimorphism. Similarly, if **B** is not symmetrical (e.g. when the covariance of trait X in females with trait Y in males is not equal to the covariance of trait Y in females and trait X in males), then one sex will respond more to sexually equivalent selection, and again dimorphism can evolve [39]. Thus, the equivalence of **G** and **B** across the sexes is important to our understanding of the ease with which sexual dimorphism and conflict weakening can evolve.

Simulations that relax some of Lande’s [2] assumptions (but assuming low mutation rates and high mutational variance) indicate that the evolution of sexual divergence can be rapid [43]. For example, if **G** and **B** evolve and population size is large (*n* = 4000), sexual dimorphism can evolve rapidly with relatively little change in the intersexual genetic correlation, and with a less intense and fairly short period of (female) maladaptation—between 6000 and 8000 generations [43]. Furthermore, the initial, steepest phase of sexual divergence can occur very quickly—100s of generations. However, with a smaller population size and/or stronger antagonistic selection, there will be slower sexual divergence and reaching equilibrium can be slow, relying on mutational input rather than standing genetic variation [43].

At present, the evolution of **B** is not well understood empirically or theoretically [30]. However, investigation of within-generation changes to **B** due to sexually antagonistic selection indicates that sexual asymmetry in the strength of selection can reduce male–female genetic covariances [30], and simulations support this inference and indicate **B** can evolve rapidly although perhaps transiently [43]. This is an area that needs additional work. There is, however, more understanding of **G** [40]. Genetic architecture can differ across populations and experimental treatments, with variations in the size and orientation of **G** [40]. Thus, **G** can also evolve rapidly over relatively short timescales (also see [44]).

Importantly, comparison of **G** across the sexes indicates that males and females may never share identical genetic variances and covariances. Estimates from three taxa (house sparrows, mealworms and white campion) show male and female matrices are completely unrelated, meaning selection would cause the sexes to evolve independently [40]. Subsequent work has also shown that while **B** can be asymmetrical and sexual differences can exist in **G**, both can constrain sex-specific evolution ([45]; also see [46]). This is consistent with models showing that conflict resolution may be slow [2] and the evolution of sex differences can generate a conflict load (maladaptation during antagonistic evolution means some individuals do not reproduce: [47]). So, while there can be significant sex differences in **G** and **B**, the sexes can still constrain each other. This is in line with some experimental work showing that generating sexual dimorphism experimentally can take hundreds of generations (e.g. [26]; but see [36]). Examination of candidate sexually antagonistic loci across the whole genome in *Drosophila melanogaster* also reveals long-term constraints on sexual dimorphism, with signatures of conflict persisting for approx. 1 Myr [48].

To summarize, there is a lack of consensus on the speed of resolution, with the precise detail of **G** and **B** likely to be taxon specific (e.g. [49,50]), and their effects also influenced by the strength of sex-specific selection. Meaningful measures of sex-specific selection and a better understanding of the genetic variance–covariance matrix will aid predictions of the evolution of sexual dimorphism. Finally, additional understanding of conflict resolution may be gained through direct assessment of mechanisms (discussed below) that cause long-term changes to both **G** and **B** to allow an uncoupling of male and female phenotypes, but there has been limited research on this to date (e.g. [51]).

### (c) Omics and resolving conflict over shared traits

**Figure 2.**
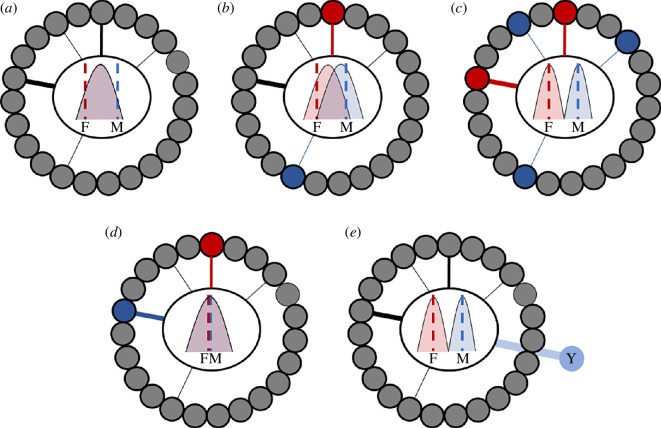
Alternative scenarios for genetic architecture and sexual dimorphism. In each panel, small circles outlined in black represent all autosomal or X-linked genes in a developmental pathway underlying a phenotype. Solid lines represent loci that contribute to variation in phenotype (genetic architecture) and the width represents effect sizes. Inner circles represent phenotype distribution in females (red) and males (blue) as well as phenotypic optima (dashed lines). Panel (*a*): representation of the traditional conceptualization of sexual conflict, where phenotypic optima differ substantially but genetic architectures are largely overlapping. Panel (*b*): male and female phenotypes can diverge towards phenotypic optima as more of the shared architecture affects only females (red circles and lines) or males (blue circles and lines). Panel (*c*): non-overlapping phenotypic distributions occur with complete decoupling of male and female architecture of a phenotype. Panel (*d*): recent work suggests that many monomorphic traits have sex differences in genetic architecture. Panel (*e*): loci on the Y chromosome (or W in ZW systems) can lead to sex-specific phenotypic distributions even when the remainder of the genetic architecture is shared.

Simulations indicate that selection should favour across-sex decoupling of traits that are under sexually antagonistic selection [43]. However, for the majority of species, most genes in the genome are present in both sexes, which suggests that the genetic architecture underlying phenotypes is largely shared (figure 2, panels *a*–*c*). Below, we detail various ways in which decoupling might occur, noting that the highly genetically interconnected (**G**) nature of the phenotype means changes in any trait may have knock-on effects elsewhere in the phenome. Therefore, reducing sexual conflict over one trait could amplify it elsewhere (e.g. [42]). Moreover, although conflict over any one shared phenotype may be resolved, conflict, in general, may be perpetual due to (i) the frequency-dependent nature of sexual selection and (ii) the changing directions and targets of sexual selection, preventing populations from fully reaching equilibrium ([52] and see [53]).

As Fisher [18] noted, sex-specific modifiers (alleles or sex hormones) could alter pleiotropy in sex-specific ways to reduce intersexual genetic correlations. For example, a sex- hormone-dependent mutation that meant an allele was only ever expressed in females would result in sex differences in pleiotropic effects and enable female and male phenotypes to diverge. In addition, any mutation that acts on the initiators of sexual differentiation themselves (e.g. sex hormones), or on their effectiveness in generating differences, would also be favoured by selection [18], and work has demonstrated how testosterone shapes genetic variances (**G**) and covariances (**B**) to facilitate sexual dimorphism [51]. In species with chromosomal sex determination, sex chromosomes trigger cascades leading to sex differentiation, but autosomal genes underpin most subsequent differentiation (e.g. [54]), clearly showing how sex-specific modification can act. Below, we discuss routes to decoupling male and female phenotypes (summarized in table 1) that can involve modifiers. We then discuss sex chromosomes in the context of conflict resolution. Finally, we highlight strategic solutions (sex-ratio adjustment) that may mask the effects of sexually antagonistic alleles. Some of this has been discussed previously (e.g. [5,7,55]), and our goal is to provide an update.

**Table 1.** Separating the sexes. Summaries of the main mechanisms enabling an uncoupling of male and female phenotypes.

main mechanisms to uncouple male- and female-shared phenotypes	description
sex-specific gene regulation	genes are expressed at different levels depending on sex (male or female)
alternative splicing	genes can be spliced differently depending on sex: different combinations of exons are joined to produce functionally different proteins
gene duplication	genes are duplicated and moved to locations where they can be regulated in a sex-specific manner
genomic imprinting	an allele is expressed according to its parent-of-origin, where maternally derived alleles are silenced (via DNA methylation) in males and paternally derived alleles are silenced in females at sexually antagonistic loci
dominance reversal	each allele at a sexually antagonistic locus is partially or completely dominant when expressed in the sex it is favoured and recessive in the sex it harms
sex chromosomes (Y or W chromosome)	the Y (or W) chromosome is a region that is limited to one sex (males or females in ZW systems), which could accumulate male-benefit alleles (or female-benefit alleles in ZW systems)
sex chromosomes (dominance effects)	the X chromosome can facilitate dominance effects, with selection favouring male-benefit alleles that are recessive and female-benefit alleles that are dominant
sex chromosomes (dosage compensation)	the sexes can differ in gene expression and protein production due to differences in sex-chromosome number
sex-ratio adjustment	a mechanism to mask the effects of sexually antagonistic alleles that does not require changes to trait genetic architecture. The sex of offspring is adjusted by the female depending on the assessment of alleles (e.g. male- or female-benefit) carried by an individual (self-assessment) or their mate

#### (i) Regulatory solutions

Regulatory decoupling (e.g. differential gene expression) can potentially alleviate conflict over shared traits and permit sexually independent evolution. Depending on the dataset, a large proportion of coding genes are expressed differently between the sexes in at least one tissue or cell type [56,57]. Sex-biased expression varies across closely related species and even populations (reviewed in [58]), suggesting it evolves rapidly, as evidenced by rapid responses to artificial selection [59,60]. Other studies have found reduced signatures of conflict associated with genome-wide sex-biased expression patterns [61,62]. Furthermore, negative associations between sex-biased expression and the occurrence of candidate sexually antagonistic loci demonstrate the potential role of regulatory decoupling in conflict resolution [48].

#### (ii) Non-regulatory solutions

Decoupling the sexes does not necessarily require dimorphism in gene regulation itself. Even for genes that are expressed at similar levels in both sexes, genetic variants may affect one sex more, or even predominantly [63], offering a potential route to decouple male and female genetic architecture and conflict reduction. Moreover, even for sexually monomorphic traits, there is evidence that genetic variants have sex-specific effects ([33], figure 2*d*). This could occur through sex-specific alternative splicing—joining of different exon combinations from the same gene to produce functionally different proteins—which occurs post-transcriptionally. Alternative splicing could be important in dimorphism generation because it avoids pleiotropic/functional constraints associated with altering gene expression levels. Although alternative splicing has been implicated in sexual dimorphism, very little is known about the phenotypic consequences and adaptive significance of sex-specific alternative splicing (reviewed in [64]). However, sex-biased expression and sex-specific alternative splicing are negatively correlated [65,66], suggesting the evolution of one might negate the need for the other. Alternative splicing may also facilitate independent evolution in the sexes [66].

#### (iii) Gene duplication

Gene duplication could also facilitate conflict resolution if paralogues adopt sex-specific function ([67,68] and see [69]). In *D. melanogaster* and other taxa, sexual dimorphism may be facilitated by male-biased expression of duplicate genes (reviewed in [69]), which is likely driven by sexual selection in males. In addition, tandem duplicates in *D. melanogaster* apparently resolve conflict over fertility traits [70]. Interestingly, although each autosomal duplicate undergoes sex-biased expression to largely resolve conflict, some antagonistic effects are still present due to residual expression in the ‘harmed’ sex. The (partial) resolution of this conflict through sex-biased expression and sequence changes occurred reasonably rapidly (approx. 2 Myr) [70].

#### (iv) Imprinting

Epigenetic parent-of-origin imprinting, whereby an allele is expressed according to its parent-of-origin, is another potential route to resolving conflict [71]. For example, males (females) that have passed the filter of sex-specific selection are more likely to carry alleles at sexually antagonistic loci that benefit their sex, and so it follows that selection should favour silencing the maternally derived allele in males (or the paternally derived allele in females) [71]. Parent-of-origin effects might also propagate to other linked genes, even those unimprinted [72], altering the expression of multiple genes at once. Imprinting is widespread and occurs in a range of taxa (e.g. [73–75]). However, empirical studies of either sex-dependent imprinting [76–79] or its role in the resolution of conflict over shared traits [80,81] are currently limited.

#### (v) Dominance reversal

Dominance reversal, where alleles are partially or completely dominant when expressed in the sex they benefit and recessive otherwise, can also act to partially mitigate conflict [82–86]. In addition, the occurrence of dominance reversal could favour the accumulation of sexually antagonistic alleles on the autosomes, instead of the X chromosome [85]. Although signatures of dominance reversal may be difficult to detect, multiple promising methods have been proposed (reviewed in [86]). Sex-dependent dominance reversal has been identified in a single large-effect locus determining salmon age at maturity [83], in a supergene that mediates trout migration tendency [87] and in a polygenic trait underlying *Drosophila* immunocompetence [88]. Seed beetles also show genome-wide dominance reversals for sexually antagonistic alleles underlying fitness [84]. As noted by Grieshop *et al.* [86], dominance reversals mitigate but do not fully resolve conflict because homozygotes will always exist to some degree in the sex where the genotype is not favoured.

#### (vi) The sex chromosomes

Owing to the sexual asymmetry in X (or Z) chromosome inheritance, theory predicts that sex-chromosome linkage can decouple female and male phenotypes and ameliorate sexually antagonistic effects (e.g. [89,90]). Specifically, male-benefit recessive alleles and female-benefit dominant alleles are expected to accumulate on the X (or Z) chromosome [90,91], with dominance effects acting to partially resolve conflict at these antagonistic loci. Complete dimorphism may then be facilitated by sex-specific modifiers that act on these sexually antagonistic loci ([90]; but see [92]).

Precise predictions regarding the accumulation of X-linked sexually antagonistic alleles are complicated by mechanisms of dosage compensation (reviewed in [93]). For example, if dosage compensation involves the inactivation of one X in females, the X-linked male recessive advantage (discussed above) no longer exists [93]. It is also worth noting that the lack of dosage compensation can itself facilitate sexual differentiation: the sexes can differ in gene expression and protein production due to differences in sex- chromosome number, potentially reducing phenotypic conflict [5]. Although there is evidence consistent with the accumulation of sexually antagonistic alleles on the X chromosome (e.g. [94–98]; but see [48]), empirical proxies of sexually antagonistic fitness variation may be biased towards detecting X-linked effects [99]. Furthermore, one unbiased test has revealed no enrichment of sexually antagonistic alleles on the human X chromosome [100], but further studies controlling for X-linked biases in a range of taxa are required.

While the role of X and Z chromosomes in conflict resolution is unclear, W and Y chromosomes provide sex-limited regions of the genome that can accumulate genes underlying sex-specific benefits [89]. Movement of male-benefit alleles to the Y chromosome (or female-benefit alleles to the W in ZW systems) can effectively decouple male and female phenotypes (figure 2*e*), and Y chromosomes continuously accumulate genes [101–104]with important male-phenotypic effects (e.g. [105–107]). For example, Y-linked additive variation impacts body-size sexual dimorphism by as much as 30% in seed beetles [108]. Studies are also increasingly demonstrating that Y-linked genes have significant epistatic effects (e.g. [109–113]), which could be important for sexual dimorphism. Further work examining the impact of sex determination systems on conflict resolution is required.

#### (vii) Sex ratio

In addition to mechanisms that resolve conflict over shared traits, offspring sex-ratio adjustment could mask effects of sexually antagonistic loci ([114]; also see [115]). For example, females might produce excess sons and fewer daughters if they mate with highly masculinized males, or they may be able to vary the sex ratio based on their own phenotype [116–121]. Male traits targeted by sexual selection, which are usually honest indicators of male quality [120,122–124], provide an obvious means for females to assess whether males carry ‘excess’ male-benefit alleles [125,126]. A single-locus model suggests that sex-ratio adjustment is likely to evolve in response to autosomal antagonistic alleles, but if antagonistic alleles are X-linked, the adjustment should be based on the homogametic genotype [114]. These strategic solutions should be kept in mind when assessing the implications of ongoing conflict over shared traits.

#### (viii) Resolution: the overall picture

There is still much progress to be made towards understanding how, and how effectively, the possible mechanisms above uncouple male and female phenotypes to resolve conflict. For example, although links between mechanisms and sexual dimorphism are often clear, impacts on sexual antagonism are rarely quantified. In addition, conflict is often studied in a univariate context, but the maintenance of sexually antagonistic fitness variation likely involves multiple traits linked by networks of interconnected genes, highlighting the need to explore conflict and its resolution in multivariate phenotypic space. It should be noted that pleiotropy and complicated gene networks may hinder conflict resolution in some instances, but they also provide many potential avenues for conflict resolution. Finally, studies across a broad taxonomic range are required, as variation in trait genetic architecture across species may impact the scope for these mechanisms to facilitate the evolution of sexually dimorphic phenotypes.

We also highlight that in the absence of complete conflict resolution, males may often be closer to fitness-maximizing phenotypes than females because of sexual selection. For example, stronger sexual selection on males may mean more masculinized phenotypes contribute disproportionately to the gene pool shared between the sexes [127,128], and there is some evidence that genomes are masculinized in populations where sexual selection is present, compared to those with experimentally enforced monogamy and no sexual selection ([59]; but also see [60,129,130]).

## 3. Empirical concerns and future directions

To fully understand conflict over shared traits, we need greater understanding of three fundamental parameters: sexually antagonistic selection, genetic variance–covariance and intersexual covariance matrices. Without documenting antagonistic selection, it is not possible to know whether conflict over a shared phenotype is present. Without understanding the genetics, we cannot determine the extent to which male and female phenotypes are decoupled or how free they are to respond to sex-specific selection. Unfortunately, it is difficult to comprehensively estimate any of these fundamental parameters with any single approach.

If our goal is to understand how sexual conflict affects natural populations, one could argue that one must go outside to study it; however, studying natural systems is difficult. For example, measures of selection are inevitably incomplete (only partial phenotypes are measured), and estimates often involve the assessment of single or small numbers of selective episodes. Thus, we may not be assessing the true targets of selection and may miss the most significant episodes of selection, including those in which sexual antagonism occurs. Long-term studies can address at least the latter issue, and as one such study shows, long-term relatively weak selection can change direction rapidly and significantly to reverse trait evolution [131]. Nonetheless, studies in natural systems that have focused on antagonistic phenotypes have revealed important aspects of sexual conflict and its resolution (e.g. [83,132]), and others have tracked allele frequency changes to reveal the interplay between selection and sexual conflict [133]. However, estimating **G** and **B** is difficult, generally requiring large sample sizes and known pedigree structures, and invariably estimates are incomplete. This is also true for field studies.

The power of experimental evolution and artificial selection in understanding conflict over shared traits has been highlighted recently [55,134], and these approaches overcome some of the difficulties mentioned above, namely the complexity of natural environments and the difficulty of multigenerational studies. Examples include sex-limited evolution, where selection on the whole phenome is limited to one sex (e.g. [135–139]), and artificial selection on specific phenotypes in one sex (e.g. [42,140–142]). However, simplistic laboratory environments that make the study of conflict more tractable may inflate sexual conflict and limit responses to it [143–145,145,146]. The very strong selection coefficients in laboratory studies may also bias estimates of genetic architecture underlying sexual conflict. Because of this, experimental evolution may be best suited to studying the rapid resolution of conflict [26,36] or the consequences of its cessation [147]. In addition, laboratory selection studies frequently involve insects and *Wolbachia* infection, which is common in these animals, can inflate estimates of sexual conflict over shared traits [148].

Linking genotype and phenotype is also crucial in understanding sexual conflict over shared traits. Evolve-and-resequence methods (e.g. [149]; discussed in [9,134]) powerfully enable the mapping of phenotypes to genome-wide sequence variation, biological processes or genetic pathways. Experimental manipulation of candidate genes and measurement of sex-specific fitness effects can also be used to verify ongoing antagonism (e.g. [70,150]). Importantly, understanding the biological significance of antagonism requires genetic manipulations in different tissue types and quantification of multivariate phenotypes over various life stages.

There is still much to learn about sexual conflict over shared traits, and critically, there are few documented loci with demonstrated sexually antagonistic effects. Detecting the genomic signatures of these loci is made difficult by the noisy nature of the signals and the complexity of selection acting on any portion of the genome [151]. However, genomic data coupled with phenotypic studies and fitness measures can identify antagonistic loci (e.g. [83,152]) and reveal the longevity of sexually antagonistic loci. In addition, experimental evolution approaches coupled with more natural levels of environmental heterogeneity [143–145,145,146] will help to disentangle factors influencing conflict. Overall, the wider implications of sexual conflict over shared traits are becoming increasingly clear, from genome architecture (e.g. [11,90,151]) to population viability (e.g. [153–155]) and even human health (e.g. [156–158]). As a result, work clarifying and deepening our understanding of this conflict is likely to deliver benefits on multiple fronts.

## Data Availability

This article has no additional data.
